# Supplementary material for: The WNT target SP5 negatively regulates WNT transcriptional programs in human pluripotent stem cells
Source: Nat Commun. 2017 Oct 18;8:1034. doi: 10.1038/s41467-017-01203-1 (PMC5647328; doi:10.1038/s41467-017-01203-1)
Supplement: Supplementary file 1 — Supplementary Information [file 41467_2017_1203_MOESM1_ESM.pdf]

a **b**

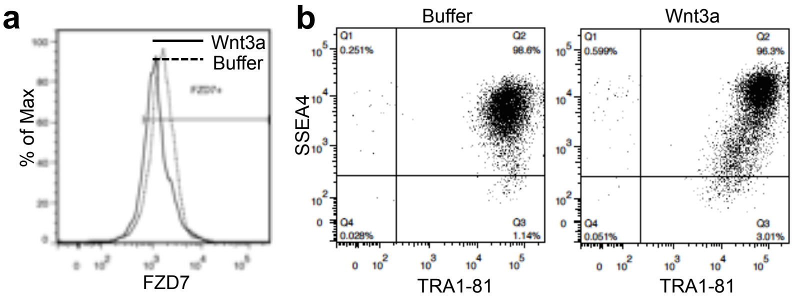

c

Gene Symbol	NCBI Gene ID	Change in 48 hr RPKM	Description
EOMES	8320	140	eomesodermin
SP5	389058	136	Sp5 transcription factor
LHX1	3975	93	LIM homeobox 1
SOX17	64321	90	SRY-box 17
MIXL1	83881	76	Mix paired-like homeobox
GATA6	2627	41	GATA binding protein 6
FOXA2	3170	40	forkhead box A2
MEIS3	56917	31	Meis homeobox 3
HHEX	3087	28	hematopoietically expressed homeobox
GSC	145258	27	goosecoid homeobox

d

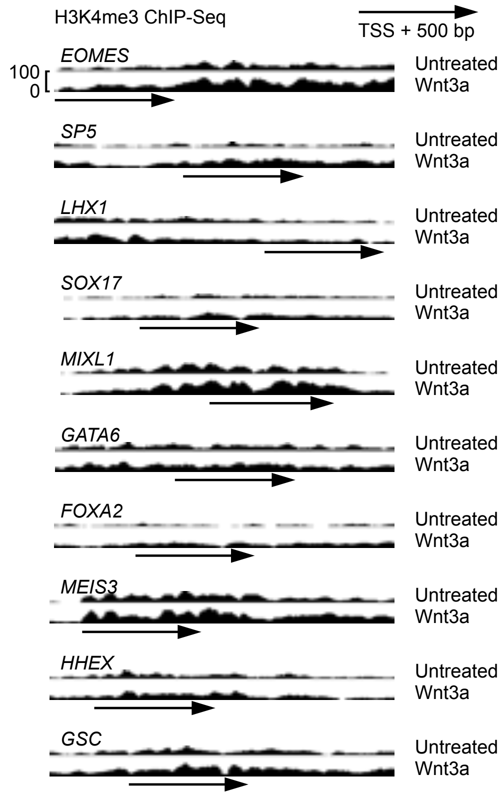

e **f** **g** **h** **i**

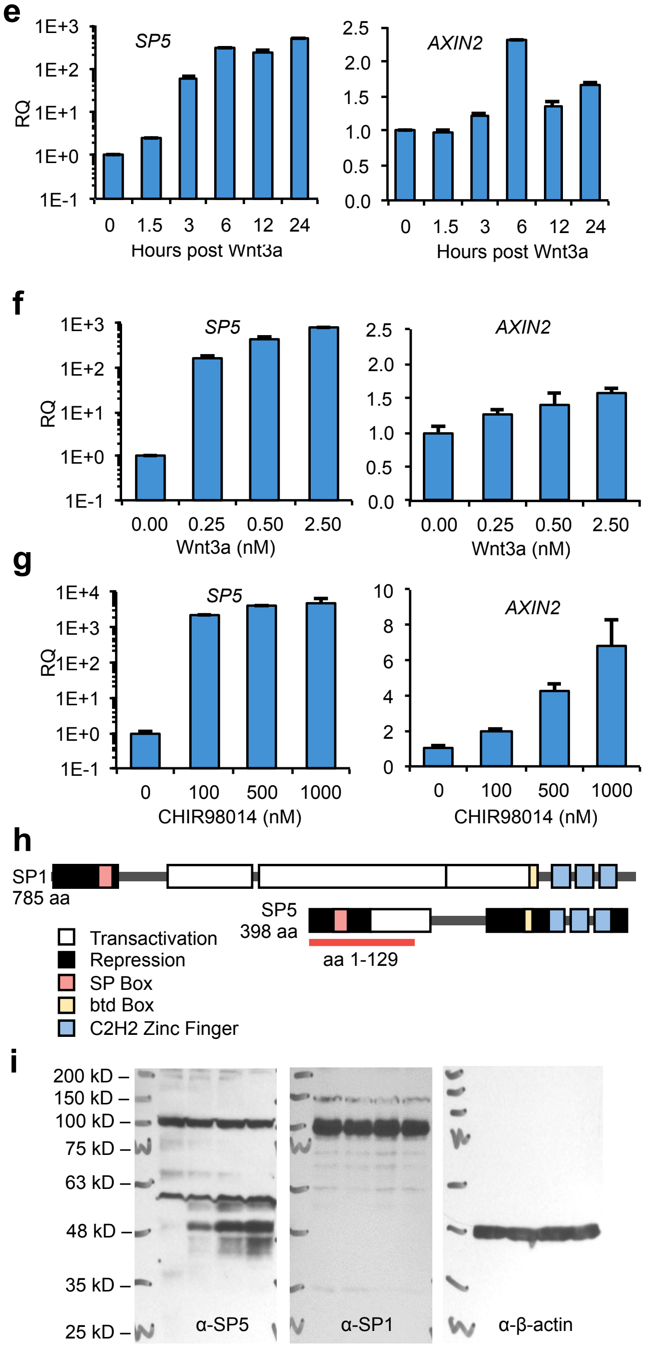

Supplementary Figure 1. Identification of SP5 as a Wnt/β-catenin target gene in hPSCs. (**a**) Flow cytometry for FZD7 shows a decline in cell surface expression of this critical pluripotency marker upon Wnt3a treatment. Cells were treated for 48 hours with 1 nM Wnt3a prior to flow cytometry. (**b**) Flow cytometry for SSEA 4 and TRA-1-81 shows a decline of these pluripotency-associated markers upon Wnt3a treatment. Cells were treated for 48 hours with 1 nM Wnt3a. (**c**) List of top 10 genes encoding transcription factors with most significant changes in expression upon Wnt3a treatment. (**d**) ChIP-Seq analysis of the histone mark K3K4me3. For each gene listed in panel A, genome browser tracks with mapped reads are shown for untreated (top) and Wnt3a-treated (bottom) hESCs. Untreated cells were incubated with an equivalent volume of Wnt storage buffer. TSS = transcriptional start site. (**e**) Timecourse of *SP5* and *AXIN2* induction. HESCs were treated with 1 nM Wnt3a for the indicated times, and total RNA was extracted and analyzed by reverse transcription quantitative PCR (qPCR). *AXIN2* expression is maximal at 6 hours and subsequently declines, whereas *SP5* induction remains steady beyond 6 hours of Wnt3a treatment. RQ = relative quantity. (**f**) Dose-response of *SP5* and *AXIN2* induction. HESCs were treated with the indicated concentrations of Wnt3a for 24 hours, and total RNA was extracted and analyzed by qPCR. RQ = relative quantity. (**g**) Expression of *SP5* and *AXIN2* in response to treatment with a GSK3 inhibitor. HESCs (H1/WA01) were treated for 24 hours with the indicated concentrations of the GSK3 inhibitor CHIR98014, and gene expression was analyzed by qPCR. RQ = relative quantity. (**h**) Schematic representation of the SP/KLF family members SP1 and SP5. Similar domains in SP1 and SP5 include the C2H2 triple zinc finger domain, SP box and buttonhead (btd) box. SP5 lacks the extensive transactivation domain found in SP1. The red line under SP5 indicates the domain used for affinity purification of a rabbit polyclonal anti-SP5 antibody raised to an N-terminal portion of SP5. aa = amino acids, btd = buttonhead, C2H2 = Zinc finger of the classical Cys_2-His_2 type, SP = specificity protein. (**i**) Uncropped images of immunoblots shown in Figure 1e. kD = kilo Daltons.

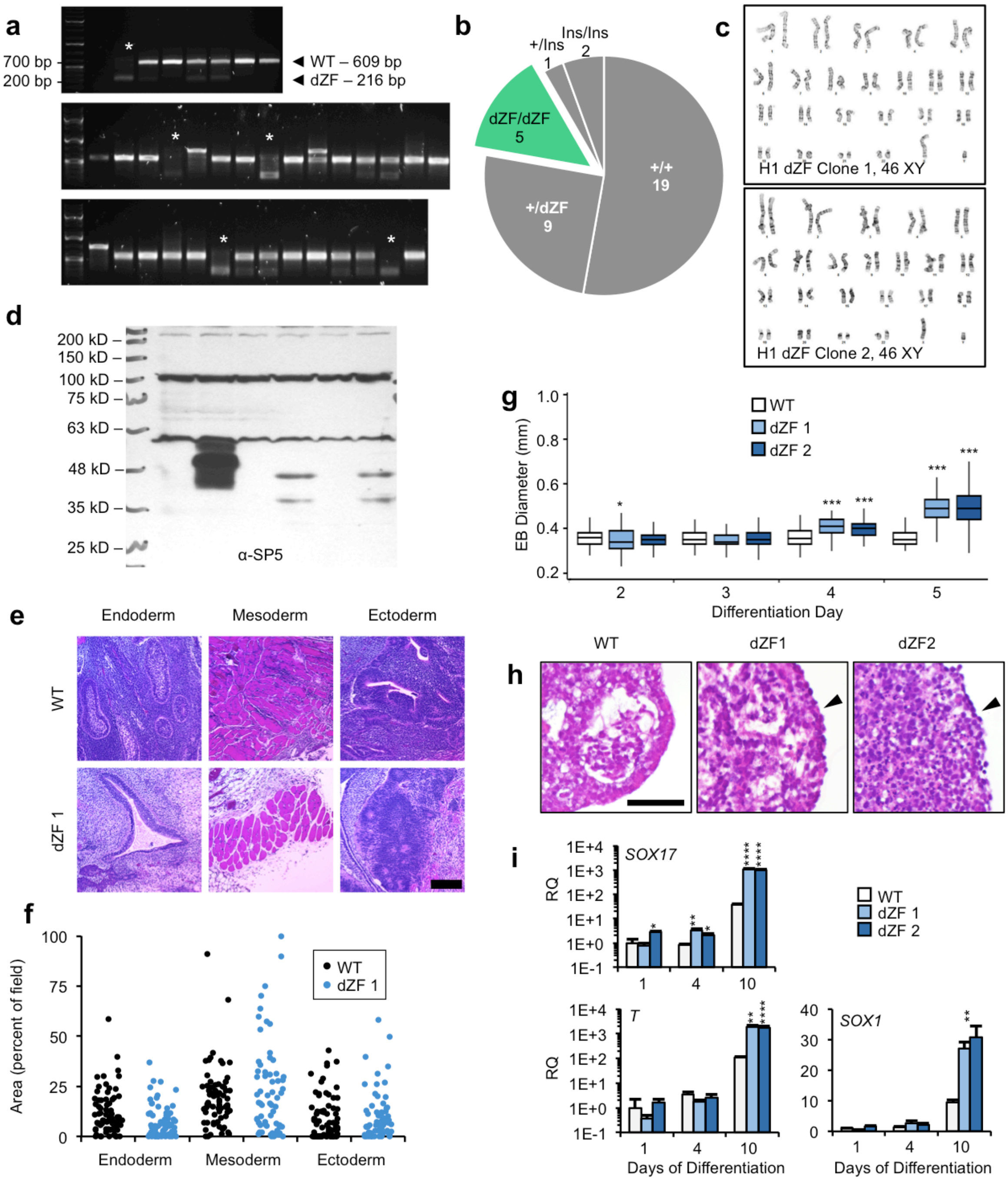

Supplementary Figure 2. SP5 is required for normal hPS cell differentiation. (**a**) Genotyping of hESC clones carrying *SP5* mutations. Genomic DNA from single cell clones of hESCs was analyzed by PCR with primers spanning the region of the *SP5* gene targeted by CRSIPR/Cas9. Clones with deletions in both alleles are indicated with an asterisk. (**b**) Quantitation of the results shown in panel a. (**c**) Karyotype analysis of *SP5* mutant cells.

Cytogenetic analysis was performed on two independent clones carrying *SP5* mutations, dZF 1 and dZF 2. None of the twenty cells of either dZF 1 and 2 exhibited chromosome aberrations. (**d**) Uncropped images of immunoblots shown in Figure 2b. kD = kilo Daltons. (**e**) Teratoma formation assay. Nude mice injected with WT or dZF1 hESCs (0.5 million cells per injection) generated tumors, which were collected after 8 weeks, stained and fixed with H&E. A total of 2 mice were injected subcutaneously into the thigh and shoulder for a total of 4 injections per cell line. Left panel, random images were taken from each sample before analysis. Scale bar = 50 µm. (**f**) Semi-quantitative analysis of teratomas. Germ layer distribution of teratomas was semi-quantified by visual inspection of H&E-stained sections in a blinded fashion. Differentiated tissue with defined areas in the teratoma were classified into ectoderm, endoderm, and mesoderm. (**g**) Size ranges of embryoid bodies. WT and two *SP5* mutant (dZF1 and dZF2) lines were differentiated for 5 days and the size of EBs was quantified daily. (**h**) Altered architecture of *SP5* mutant EBs. Day 10 EBs were fixed, parafin embedded, sectioned and stained with H&E. *SP5* mutant EBs exhibit abnormally rough outer cell layers (arrowheads). Scale bar = 50 µm. (**i**) Altered gene expression in *SP5* mutant EBs. RNA was isolated from EBs at the indicated days after initiation of differentiation and analyzed by qPCR for expression of markers indicative of endoderm (*SOX17*), mesoderm (*T*) and ectoderm (*SOX1*). (ns - not significant; * - $p < 0.05$; ** - $p < 0.01$; *** - $p < 0.001$; **** - $p < 0.0001$).

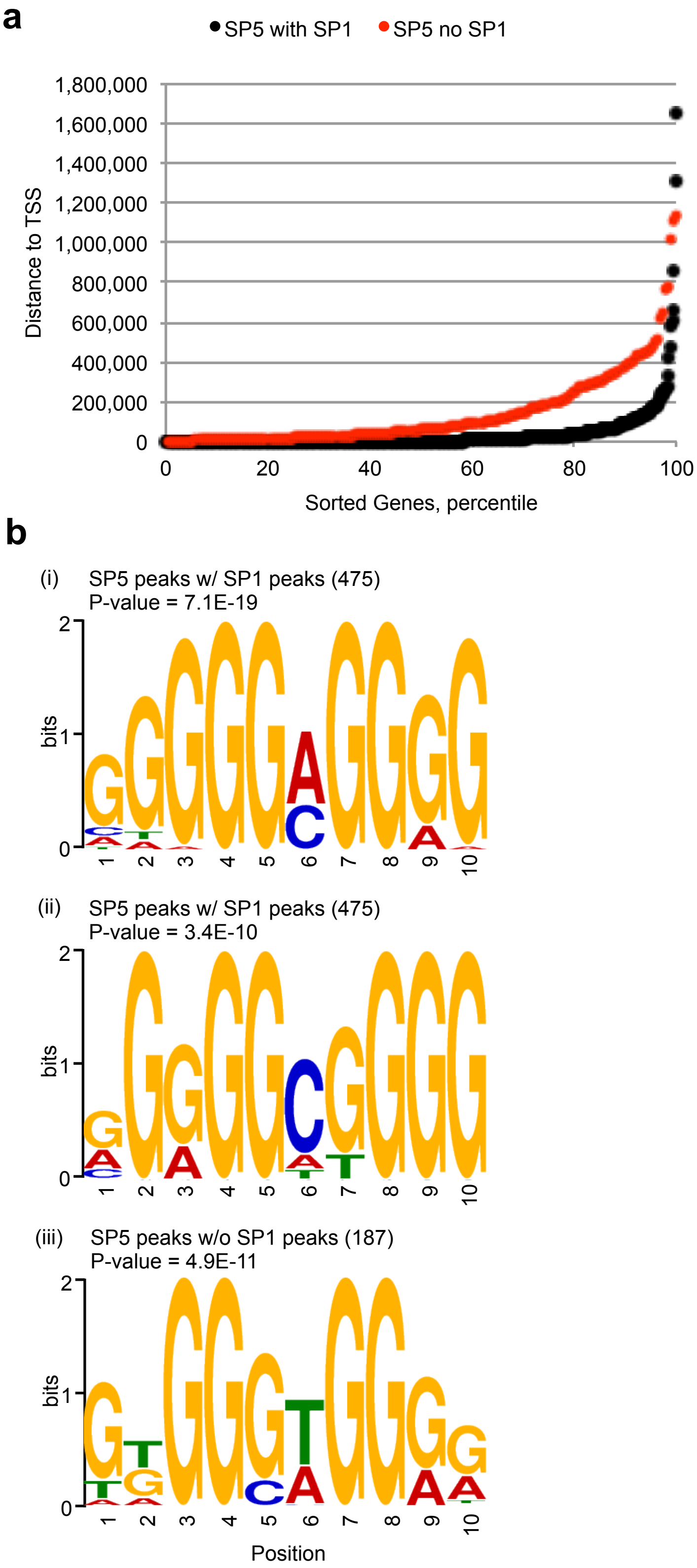
a
SP5 with SP1
SP5 no SP1
1,800,000
1,600,000
1,400,000
1,200,000
1,000,000
800,000
600,000
400,000
200,000
0
Distance to TSS
0
20
40
60
80
100
Sorted Genes, percentile
b
(i) SP5 peaks w/ SP1 peaks (475)
P-value = 7.1E-19
bits
(ii) SP5 peaks w/ SP1 peaks (475)
P-value = 3.4E-10
bits
(iii) SP5 peaks w/o SP1 peaks (187)
P-value = 4.9E-11
bits
Position

Supplementary Figure 3. SP5 ChIP-Seq analysis. (**a**) Distance distribution of SP5 peaks from TSS. (**b**) Motif analysis of SP5 peaks. SP5 binding events were analyzed for overrepresented sequence motifs using MEME. (i) and (ii) The second and third most over-represented motifs for the 475 SP5 peaks with an overlapping SP1 peak closely match the GC box. (iii) The second most over-represented motif for the 187 SP5 peaks lacking an overlapping SP1 peak is clearly divergent from the canonical GC box.

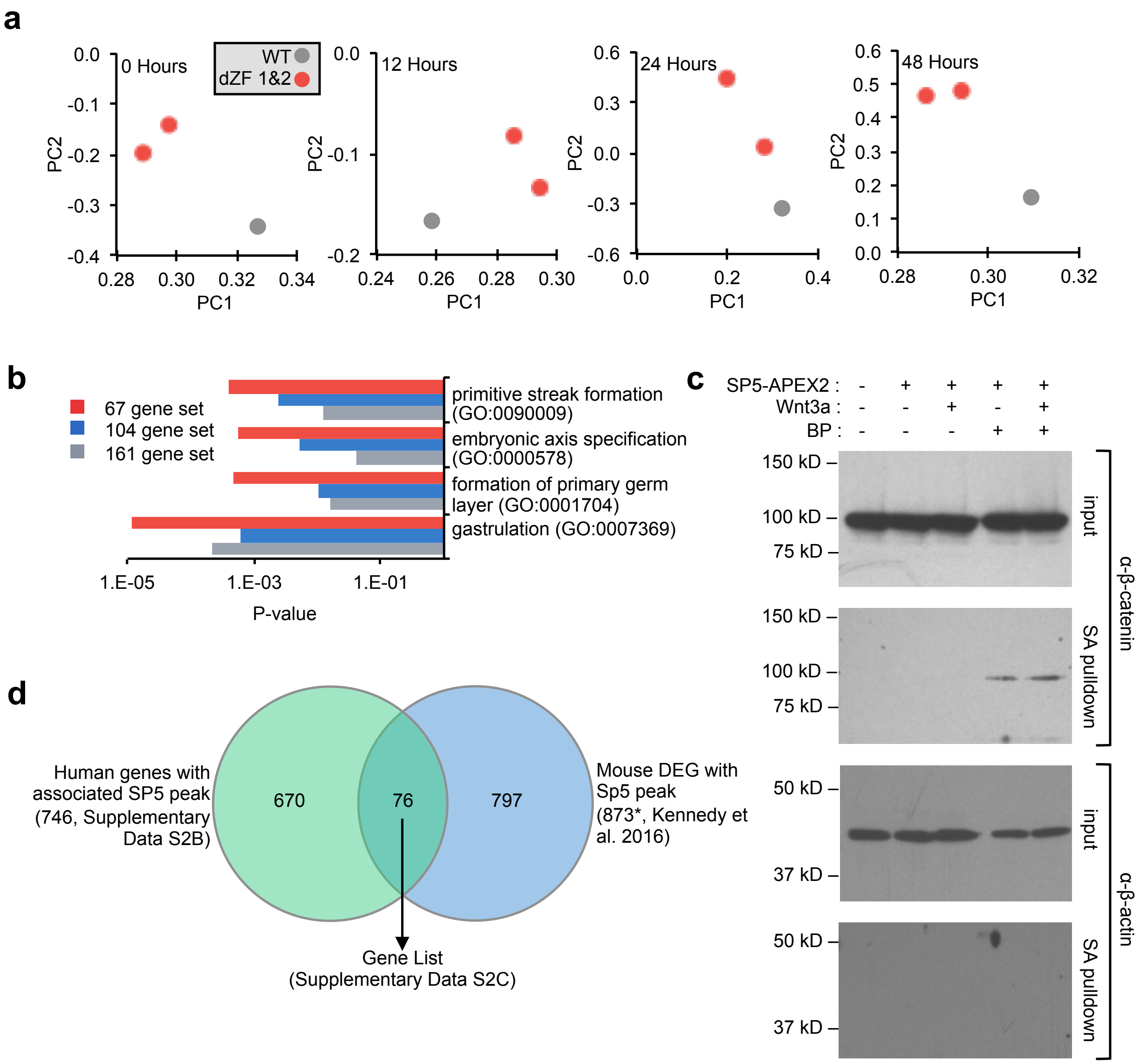

Supplementary Figure 4. De-regulation of gene expression in *SP5* mutant hPSCs. (**a**) Principal component analysis of WT and dZF gene expression data. WT cells (gray dots) generally cluster further from dZF 1 and dZF 2 (red dots), indicating that the mutant cells are more alike to each other than to wild type at each timepoint. (**b**) SP5 peaks are located near genes that are significantly enriched for genes associated with primitive streak formation. This bar graph provides p-values to the graph shown in Fig. 4d. (**c**) Proximity ligation demonstrates SP5-β-catenin interaction. HEK293 cells were transiently transfected with an expression vector carrying an APEX2-SP5 fusion gene. 24 hours post-transfection cells were treated with Wnt3a.

48 hours post-transfection cells were treated with Biotin-phenol (BP) and hydrogen peroxide. Whole cell lysates (input) and Streptavidin precipitated proteins (SA pulldown) were analyzed by immunoblotting for β-catenin and β-actin. (**d**) Overlap of mouse and human genes with an associated SP5 peak. List of human genes and list of genes in common between mouse and human genes sets are provided in Supplementary Data 2. *The list of published 892 genes published by Kennedy was trimmed to delete mouse gens that lack a human homolog.

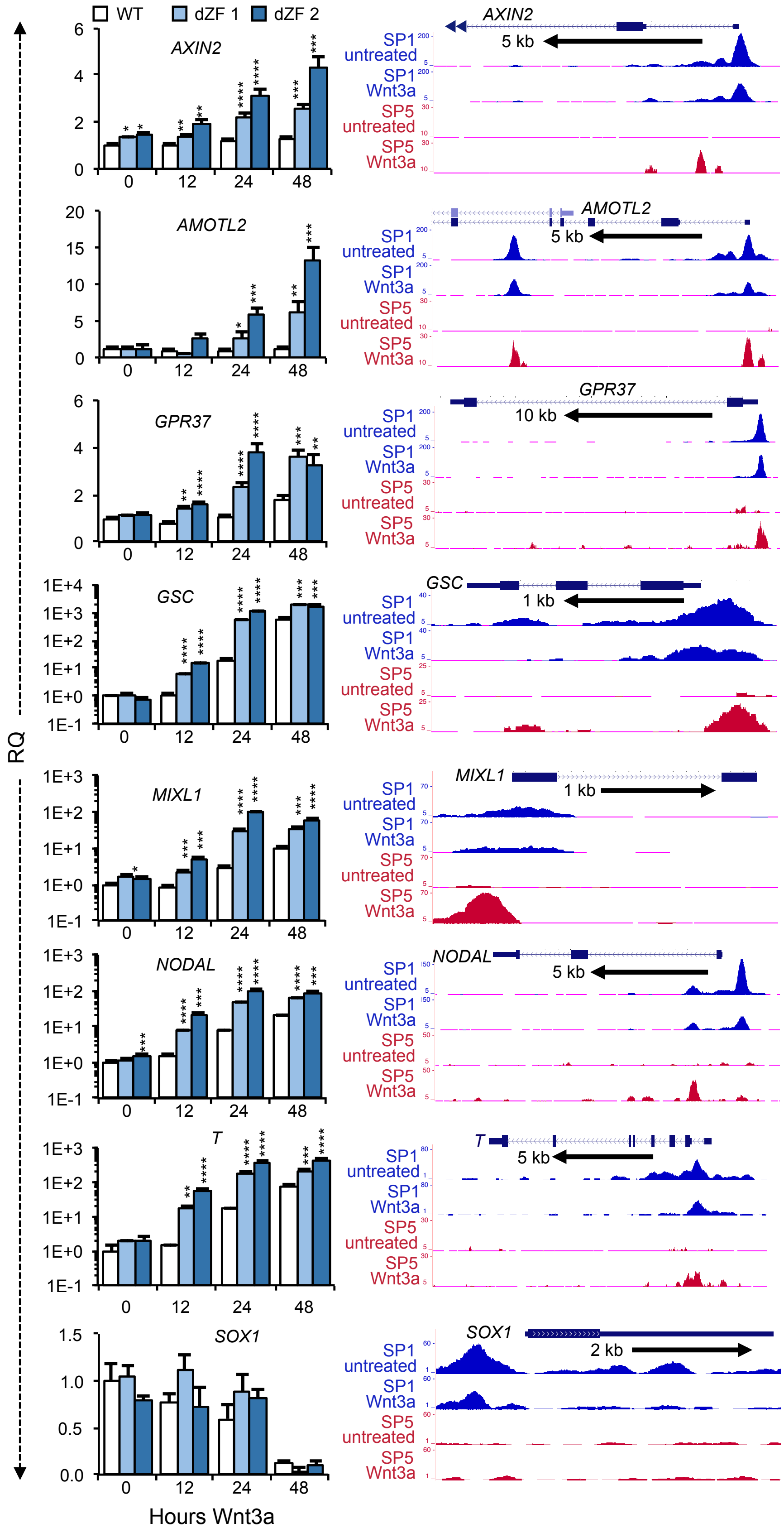

WT
dZF 1
dZF 2
RQ
AXIN2
AMOTL2
GPR37
GSC
MIXL1
NODAL
T
SOX1
0
12
24
48
Hours Wnt3a
SP1 untreated
SP1 Wnt3a
SP5 untreated
SP5 Wnt3a
5 kb
10 kb
1 kb
2 kb

Supplementary Figure 5. Integrated analysis of SP5 mutant RNA-Seq and SP5 ChIP-Seq Identifies Critical Targets of SP5 in hPSCs. WT and dZF mutant hESCs were treated with 1 nM Wnt3a for the indicated times and RNA was isolated and analyzed by qPCR for expression of *AXIN2*, *AMOTL2*, *GPR37*, *GSC*, *MIXL1*, *NODAL*, *T* and *SOX1*. Genome browser tracks are provided to illustrate the increase of SP5 binding near the transcriptional start site of each gene. *SOX1*, which lacks an associated SP5 peak, shows no differences in expression upon Wnt3a treatment in WT and mutant cell lines. (ns – not significant; * - $p < 0.05$; ** - $p < 0.01$; *** - $p < 0.001$; **** - $p < 0.0001$).

a

Cell line	Description	*AXIN2* RQ	*AXIN2* SEM	*SP5* RQ	*SP5* SEM
BT549	Mammary carcinoma	2.72	0.14	2.08	0.69
H1/WA01	Human embryonic stem cell	1.56	0.08	797.7	51.26
H1299	Lung carcinoma	28.61	0.94	2.25	0.08
HEK293T	Embryonic kidney	1.87	0.18	1.85	0.48
HeLa	Cervical adenocarcinoma	16.76	1.20	2.04	0.20
hNPC	Neural progenitor cell derived from hESCs	8.28	0.44	174.8	10.62
K562	Lymphoblast, CML	1.38	0.10	0.54	0.06
MDA-MB-231	Mammary adenocarcinoma	5.61	0.31	1.39	0.05
MPC	Mesodermal progenitors derived from hESCs	0.83	0.08	1.19	0.11
NCCIT	Pluripotent embryonal carcinoma	17.92	1.78	1555	175.3
OVCAR3	Ovarian adenocarcinoma	18.16	0.86	3.60	0.29
OVCAR4	Ovarian adenocarcinoma	2.54	0.12	2.13	0.32
PA1	Ovarian teratocarcinoma	2.61	0.11	24.74	1.67
SKOV3	Ovarian adenocarcinoma	9.79	0.27	1.55	0.09
SW480	Colorectal adenocarcinoma	1.57	0.07	1.73	0.22
T47D	Mammary carcinoma	131.9	11.45	5.29	0.53

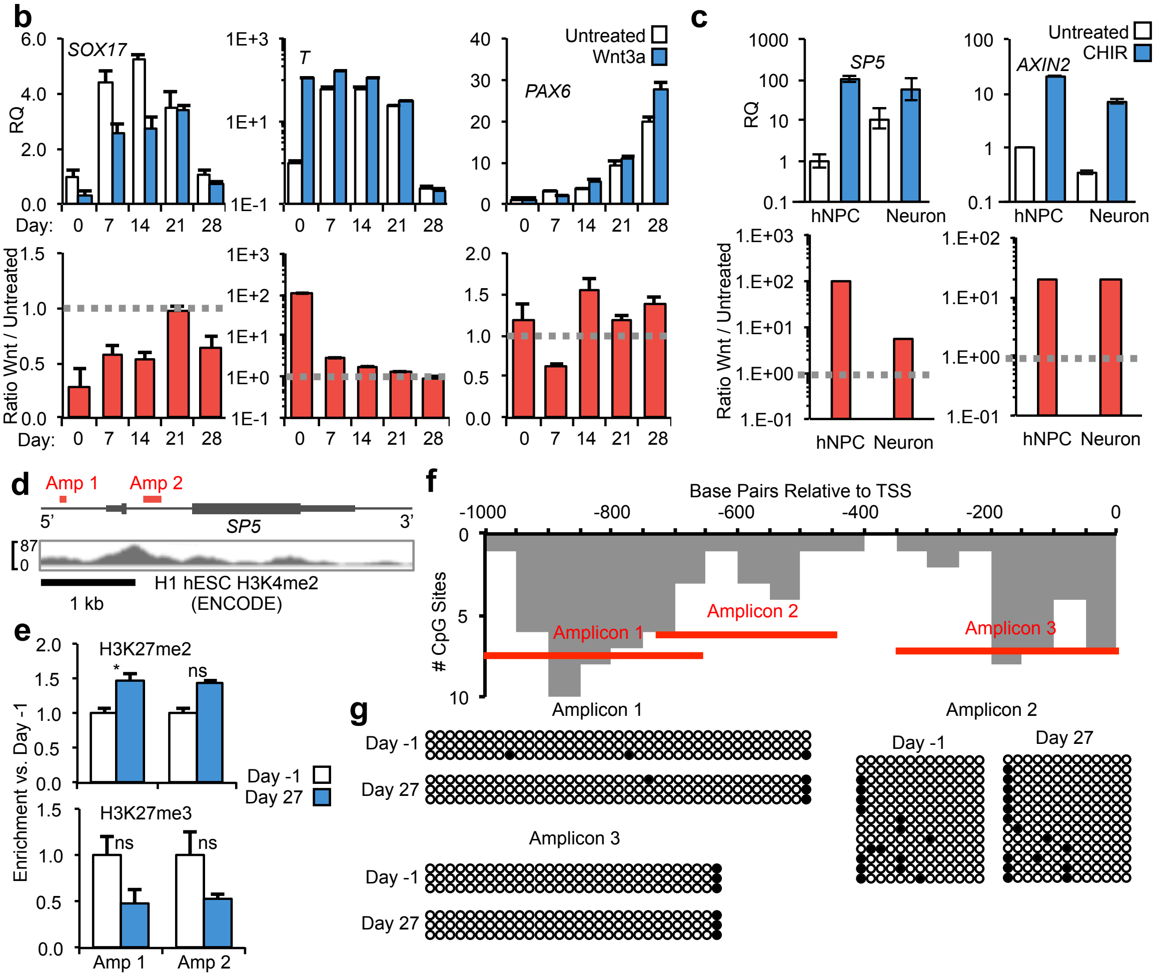

Supplementary Figure 6. Loss of SP5 induction following prolonged differentiation. (**a**) Induction of *SP5* and *AXIN2* in multiple cell lines. The indicated cell lines were treated with

Wnt3a for 24 hours and *SP5* and *AXIN2* expression was determined by qPCR. RQ = relative quantity, SEM = standard error of the mean. (**b**) Expression of early germ layer markers upon Wnt stimulation. HESCs (H1/WA01) were treated for 24 hours with Wnt3a or buffer (untreated), RNA was harvested on the indicated day post-differentiation and analyzed by qPCR for levels of the germ layer markers *SOX17* (endoderm), *T* (mesoderm) and *PAX6* (ectoderm). The bottom graphs represent the ratios of relative gene expression in Wnt3a treated versus untreated cells. (**c**) Induction of *SP5* and *AXIN2* in human neural progenitor cells (NPCs) and derivative neurons. Graphs represent the ratio of relative *SP5* and *AXIN2* expression in CHIR98014 (CHIR) treated versus untreated in NPCs and neurons. (**d**) Schematic of *SP5* locus. The positions of the two amplicons (Amp) used in Supplementary Fig. 6e for PCR of the immunoprecipitated chromatin are indicated. Also shown is the sequencing coverage of the histone mark H3K4me2 across the *SP5* locus in H1 cells (source: ENCODE). (**e**) ChIP-PCR analysis of the *SP5* promoter region. Cross-linked chromatin was isolated from cells prior to differentiation (Day -1) and on Day 27 of differentiation and immunoprecipitated with antibodies to either the histone mark H3K27me2 or H3K27me3. Enrichment of immunoprecipitated chromatin was quantified by PCR of two regions of the SP5 promoter, amplicons (Amp) 1 and 2, which are depicted in Supplementary Fig. 6d. Only subtle differences are observed for H3K27me2 and H3K27me3 occupancy in differentiated cells relative to undifferentiated cells. (ns – not significant; * - $p < 0.05$). (**f**) Schematic of upstream regulatory region of *SP5*. The positions of the three amplicons used for methylation analysis are shown in red. The graph provides the number of CpG sites at the indicated positions relative to the transcriptional start site (TSS) of *SP5*. (**g**) Methylation analysis of the SP5 promoter region. Genomic DNA from undifferentiated and 28 day differentiated cells was analyzed for CpG methylation using bisulfite sequencing. The schematics indicate the methylation state of the upstream regulatory region across Amplicons 1, 2 and 3. No differences in methylation state are observed in the examined upstream regions.

SUPPLEMENTARY TABLES

Supplementary Table 1: List of primers

QPCR primers

18S	GTAACCCGTTGAACCCCATT	CCATCCAATCGGTAGTAGCG
AMOTL2	GTCCCGTCGATGGGTTTAGG	GCTTCTTTGGCTTGCACACA
AXIN2	TATCCAGTGATGCGCTGACG	CGGTGGGTTCTCGGGAAATG
CXCR4	ACTACACCGAGGAAATGGGCT	CCCACAATGCCAGTTAAGAAGA
FOXA2	GGAGCAGCTACTATGCAGAGC	CGTGTTCATGCCGTTCATCC
GAPDH	ACAACTTTGGTATCGTGGAAGG	GCCATCACGCCACAGTTTC
GPR37	CCGATCTTCCGCTATCACCC	TGCTGCTGAGAGTTAGGCAC
GSC	CGGGACACTTGCCCGTATTA	CCTCCCGGCTCTGTACACTA
MIXL1	GGATCCAGCTTTTATTTTCTCCCC	GGCCTAGCCAAAGGTTGGAA
NANOG	TTTGTGGGCCTGAAGAAAACT	AGGGCTGTCCTGAATAAGCAG
NODAL	CCCAAGCAGTACAACGCCTA	TGCATGGTTGGTCGGATGAA
PAX6	TTGAAAAGGGAACCGTGGCT	TCACTGGCCCATTAGCGAAG
POU5F1	CTTGAATCCCGAATGGAAAGGG	GTGTATATCCCAGGGTGATCCTC
RPL37A	ATTGAAATCAGCCAGCACGC	GATGGCGGACTTTACCGTGA
SOX1	GGCTTTTGTACAGACGTTCCC	AACCCAAGTCTGGTGTCAGC
SOX17	GTGGACCGCACGGAATTTG	GGAGATTCACACCGGAGTCA
SP5	TCGGACATAGGGACCCAGTT	CTGACGGTGGGAACGGTTTA
T	CTATTCTGACAACTCACCTGCAT	ACAGGCTGGGGTACTGACT

Primers for SP5 promoter ChIP-qPCR

Amplicon 1	TGGAAGCCGAAGGCAGATTT	AAAGATTTGCACCGACGCAG
Amplicon 2	GGATCCGGGATTGTTCGGAG	CGTGGTCAGTAAGAGTCGGG

Primers for SP5 promoter methylation bisulfite sequencing

Amplicon 1	TATTTTGTAAAAGTTGTGGTTTGA GGAAAATTTTTAGG	AACTTTAAAACRACAAAAAATAT AACTCTCCCC
Amplicon 2	TATTATAGGAGTTTTGTGGATTTA AAGGATTTG	AACTACRACTTACAAACCCCTAA CTAC
Amplicon 3	GGYGAGGGTGTAGGGTGTGTAA GTAAATATAGG	ACACAAATAATTAAATTTTCCTAA TCAAAAAAAACC
