# Supplementary material for: The WNT target SP5 negatively regulates WNT transcriptional programs in human pluripotent stem cells
Source: Nat Commun. 2017 Oct 18;8:1034. doi: 10.1038/s41467-017-01203-1 (PMC5647328; doi:10.1038/s41467-017-01203-1)
Supplement: Supplementary file 3 — Description of Additional Supplementary Files [file 41467_2017_1203_MOESM3_ESM.pdf]

Description of Additional Supplementary Files

File Name: Supplementary Data 1
Description: Expression data for hPSCs treated with Wnt3a Tab S1A. RPKM values for 511 genes with significant fold changes in expression in response to Wnt3a.

File Name: Supplementary Data 2
Description: List of SP5 binding sites.

File Name: Supplementary Data 3
Description: Expression data for WT, dZF1 and dZF2 hPSCs treated with Wnt3a. RPKM values for 511 genes with significant fold changes in expression in response to Wnt3a in wildtype and SP5 mutant cells.

File Name: Supplementary Data 4
Description: List of gene sets A-D. Each tab provides the list genes of gene sets A, B, C and D in Fig. 4.
